# Discomfort and intolerance during 24-hour multichannel intraluminal impedance-pH monitoring

**DOI:** 10.1017/S0022215126105192

**Published:** 2026-08

**Authors:** Hyun Jee Lee, Seok-Hwan Chung, Seok-Ju Yoo, Soo-Yeon Kim, Jun-Yuel Yoo, Su Il Kim, Young Chan Lee, Young-Gyu Eun

**Affiliations:** Department of Otolaryngology–Head & Neck Surgery, Kyung Hee University School of Medicine, Kyung Hee University Medical Center, Seoul, Korea

**Keywords:** laryngopharyngeal reflux, visual analog scale, surveys and questionnaires

## Abstract

**Objective:**

To investigate pain, discomfort, tolerance and complications during 24-hour multichannel intraluminal impedance-pH monitoring.

**Methods:**

A prospective observational study at an academic out-patient clinic including 55 patients undergoing 24-hour multichannel intraluminal impedance-pH monitoring. Patient experience was measured using self-reported questionnaires at four time points: pre-insertion, post-insertion, before sleep and post-procedure. Anxiety, pain, discomfort, tolerance and other symptoms were assessed using visual analogue scale (VAS) scores. After treatment, the decision regret scale survey was administered.

**Results:**

Anxiety decreased after catheter insertion. Discomfort peaked immediately after insertion and declined over time, while intolerance remained relatively stable. Foreign body sensation showed the highest VAS score. The proportion of participants unwilling to undergo the examination again was high, but patients tended to have a positive perception of 24-hour multichannel intraluminal impedance-pH monitoring after treatment.

**Conclusion:**

Although 24-hour multichannel intraluminal impedance-pH monitoring has been reported to be uncomfortable and difficult to tolerate, most patients were satisfied with their decision to undergo the test.

## Introduction

Laryngopharyngeal reflux (LPR) is the retrograde flow of gastric or duodenal contents into the pharynx and larynx, resulting in irritation and inflammation of the upper aerodigestive tract.^1^ Interestingly, the incidence of LPR has increased dramatically over time.^2^ It has been reported in up to 10 per cent of patients presenting to an otolaryngology office,^3^ and more than 50 per cent of patients with hoarseness have been found to have reflux-related disease.^4^ Patients with LPR often present with hoarseness; however, almost two-thirds do not report heartburn. Other throat symptoms, such as foreign body sensation, dysphagia, chronic throat clearing and cough, are often associated with LPR.^5^ The physical findings of LPR can range from mild, isolated oedema and/or erythema of the arytenoid cartilage area to diffuse laryngeal oedema and hyperaemia, with granuloma formation and airway obstruction.^6^

Although LPR is common, its diagnosis may be difficult because symptoms are non-specific and laryngeal findings are not always associated with symptom severity. Failure to recognise LPR may result in prolonged symptoms and delayed healing,^1^ whereas overdiagnosis can lead to unnecessary costs and missed diagnoses. A diagnosis of LPR may be established by asking patients about specific symptoms, laryngoscopy or 24‐hour double-probe pH monitoring, which remains the standard diagnostic test. As there is no consensus on the optimal test for diagnosing LPR, the majority of clinicians depend on clinical symptoms and the response to empirical therapy with proton pump inhibitors (PPIs) to make the diagnosis.^7^

The practice of 24-hour multichannel intraluminal impedance-pH monitoring is considered the ‘gold standard’ for diagnosing LPR.^8^^–^^11^ However, the invasive nature of this method limits widespread use. Although 24-hour multichannel intraluminal impedance-pH monitoring is known to be uncomfortable and invasive, there is a lack of research about this. We therefore aimed to investigate patient pain, discomfort, tolerance and complication during 24-hour multichannel intraluminal impedance-pH monitoring.

## Materials and methods

### Subjects and study design

This prospective, single-centre study was conducted at a tertiary academic medical centre and approved by the Institutional Review Board of Kyung Hee University Medical Center. The study population consisted of patients who underwent 24-hour multichannel intraluminal impedance-pH monitoring. The exclusion criteria included age under 18 years, pregnancy, insufficient comprehension of the questionnaire or procedure, and refusal to participate in the study.

Patient experience was measured using self-reported questionnaires at four time points: before catheter insertion (pre-insertion), immediately after catheter insertion (post-insertion), before sleep during monitoring (pre-sleep) and after catheter removal (post-procedure). A pre-insertion questionnaire was administered prior to the start of the examination. The post-insertion questionnaire was completed immediately after the catheter placement. During the monitoring period, a pre-sleep questionnaire was administered before the patient went to bed. The post-procedure questionnaire was completed after the catheter was removed. The otolaryngologist who conducted the examinations completed the physician survey.

In the physician evaluation section, patient demographics, including age, sex and examination date, were recorded. Physicians also reported whether the patient had completed the examination in yes or no format. They documented any complications during the examination, such as epistaxis, mucosal injury or syncope (Supplementary Table S1).

In the patient questionnaire, anxiety was assessed at two time points, before catheter insertion (pre-insertion) and immediately after insertion (post-insertion), using a visual analogue scale (VAS) ranging from 0 (no anxiety) to 10 (worst possible anxiety) (Supplementary Tables S2 and S3). Pain, discomfort and intolerance were assessed using VAS scores at three time points: immediately after catheter insertion (post-insertion), before sleep during the monitoring period (pre-sleep) and after catheter removal (post-procedure) (Supplementary Tables S4 and S5).

In the post-insertion questionnaire, patients were also asked about specific symptoms, including nasal pain, throat pain, foreign body sensation, nausea, vomiting and dyspnoea. Finally, in the post-procedure questionnaire, patients were asked whether they would undergo the examination again if necessary, with responses recorded as yes or no (Supplementary Table S5).

After completing the 24-hour multichannel intraluminal impedance-pH monitoring and receiving appropriate treatment based on the results, the patients were asked to complete an additional survey assessing their decision regret using the validated five-item Decision Regret Scale^12^ (Supplementary Table S6). Each question was assessed by choosing one of five options: strongly disagree, disagree, neither agree nor disagree, agree or strongly agree.

### Data collection and statistical analysis

The paper surveys were entered into a spreadsheet. Data were analysed descriptively and statistically using SPSS version 20.0 (IBM Corp., Armonk, NY, USA). The Friedman and Wilcoxon signed-rank tests were applied when appropriate. Statistical significance was set at *p* less than 0.05.

## Results

### Participant characteristics and procedure outcomes

A total of 55 patients underwent 24-hour multichannel intraluminal impedance-pH monitoring. The mean age was 55.4 years (range, 18–75 years) and there were 30 male patients and 25 female patients. All the patients completed the examination without any failure. There were two cases of mucosal injury resulting from the examination (Table 1).

**Table 1.** Demographics and procedure outcomes Table 1 long description.

Characteristics	Value
Age (mean (range); years)	55.4 (18−75)
Female gender (*n* (%))	25 (45.5)
Examination completion rate (%)	100 (55 of 55 completed)
Examination failure (%)	0
Complication	Two mucosal injuries

### Visual analogue scale scores

The median anxiety score decreased from 5.0 (interquartile range, 3.0–7.0) before catheter insertion to 3.0 (interquartile range, 0–5.0) immediately after insertion. This reduction was statistically significant (*p* = 0.001). Self-reported VAS scores for pain, discomfort and intolerance were recorded at three time points: post-insertion, pre-sleep and post-procedure. For pain, the median VAS score was 4.0 (interquartile range, 2.0–6.0) immediately after catheter insertion (post-insertion), 4.0 (interquartile range, 2.0–6.0) before sleep (pre-sleep) and 2.0 (interquartile range, 1.0–5.0) after catheter removal (post-procedure). Pain scores were not significantly different between post-insertion and pre-sleep (*p* = 0.458) but significantly decreased from pre-sleep to post-procedure (*p* = 0.009).

For discomfort, the VAS scores were 6.0 (interquartile range, 3.0–7.0) at post-insertion, 5.0 (interquartile range, 3.0–7.0) at pre-sleep and 3.0 (interquartile range, 1.0–5.5) at post-procedure. No significant difference was observed between post-insertion and pre-sleep (*p* = 0.403), but a significant decrease was observed from pre-sleep to post-procedure (*p* = 0.013). For intolerance, the scores were 5.0 (interquartile range, 2.5–6.0) at post-insertion, 5.0 (interquartile range, 2.0–7.0) at pre-sleep and remained at 5.0 (interquartile range, 2.0–6.0) at post-procedure. The intolerance scores did not differ significantly across the three time points (*p* = 0.682). Among the three symptoms assessed, both pain and discomfort showed significant reductions from pre-sleep to post-procedure, whereas intolerance remained unchanged throughout the observation period.

Local and specific symptoms were evaluated immediately after catheter insertion using a VAS. Among the reported symptoms, foreign body sensation showed the highest median score of 4.0 (interquartile range, 2.0–6.0), followed by throat pain 3.0 (interquartile range, 0–4.5) and nasal pain 2.0 (interquartile range, 0–4.0). Nausea 0 (interquartile range, 0–3.0), dyspnoea 0 (interquartile range, 0–2.0) and vomiting 0 (interquartile range, 0–0.5) were also reported, although they had lower median scores.

When asked about their willingness to undergo testing again if necessary, 56.4 per cent of the patients who underwent 24-hour multichannel intraluminal impedance-pH monitoring answered yes. The question was included in the post-procedure survey administered after catheter removal and responses were collected from all participants who completed the examination.

### Follow-up assessment of decision regret scale after treatment

Of the 55 patients who completed the 24-hour multichannel intraluminal impedance-pH monitoring and subsequent treatment, 33 responded to a follow-up survey assessing their retrospective perception of the decision to undergo testing. These patients were asked to complete a validated five-item Decision Regret Scale (Supplementary Table S6). Each question was assessed by choosing one of five options: strongly disagree, disagree, neither agree nor disagree, agree or strongly agree.

In response to the question of whether undergoing the 24-hour multichannel intraluminal impedance-pH monitoring was the right decision, 64 per cent of patients provided positive responses (strongly agree, 21 per cent; agree, 43 per cent). When asked if they regretted undergoing the test, 76 per cent of the patients strongly disagreed (30 per cent) or disagreed (46 per cent). Regarding whether they would choose to undergo the test again if necessary, 27 per cent responded positively (strongly agree, 12 per cent; agree, 15 per cent). Only 3 per cent reported that the test had caused harm (strongly agree, 0 per cent; agree, 3 per cent). When asked if they thought undergoing the test was a wise choice, 61 per cent of patients showed a positive response (strongly agree, 21 per cent; agree, 40 per cent) (Table 2).

**Table 2.** Decision Regret Scale Table 2 long description.

Question	Strongly agree (%)	Agree (%)	Neither agree nor disagree (%)	Disagree (%)	Strongly disagree (%)
1. Right decision	21	43	15	15	6
2. Regret decision	12	9	3	46	30
3. Same choice	12	15	3	30	40
4. Did me harm	0	3	3	61	33
5. Wise decision	21	40	15	18	6

## Discussion

Laryngopharyngeal reflux is characterised by chronic laryngopharyngeal symptoms such as hoarseness, vocal fatigue, excessive throat clearing, foreign body sensation, cough and post-nasal drip, as well as laryngoscopic signs such as erythema, oedema, ventricular obliteration, post-cricoid hyperplasia and pseudosulcus change.^13^ The quality of life of LPR patients is generally poor and they often experience more severe somatic anxiety symptoms.^14^^–^^17^

Diagnosis and management are challenging because of the non-specific symptoms and laryngoscopic findings. Traditionally, empirical treatment with PPIs once or twice daily has been considered a pragmatic therapeutic strategy, with reflux testing recommended for those refractory to high-dose PPIs.^1^^,^^18^^,^^19^ However, this approach has recently been challenged by the use of upfront 24‐hour multichannel intraluminal impedance-pH monitoring prior to anti-reflux therapy to minimise the cost.^20^ When patients presenting with LPR symptoms were evaluated with 24-hour multichannel intraluminal impedance-pH monitoring, only 68.3 per cent demonstrated true hypopharyngeal reflux, while 31 per cent showed no reflux.^21^ This suggests that upfront testing may be necessary to reduce unnecessary PPI treatment. Although 24-hour multichannel intraluminal impedance-pH monitoring is considered the best diagnostic method, it is not widely used.^8^^,^^10^^,^^22^ Its diagnostic performance has been well established, but patient-centred aspects of the test,^23^ including discomfort, tolerance and overall experience, remain underexplored. We therefore sought to understand the extent of discomfort and intolerance experienced by patients in this study.

The results of our study addressed several important aspects of LPR diagnosis, particularly pain, discomfort, tolerance and complications during 24-hour multichannel intraluminal impedance-pH monitoring. We observed a low incidence of complications and absence of examination failures, underscoring the safety profile of the technique and suggesting that it can be performed with relatively low risk despite its characterisation as an invasive test. In addition, despite experiencing discomfort and intolerance during the procedure, most patients ultimately accepted the test and reported a low level of regret, as reflected in the Decision Regret Scale. Many patients considered the examination a wise choice and expressed their willingness to undergo it again if necessary. These findings suggest that patients are more likely to accept a test if its clinical value outweighs the associated physical discomfort.

We also found a distinct time-related pattern in patient-reported symptoms during the 24-hour monitoring: discomfort gradually decreased, whereas intolerance was sustained at a similar level throughout the procedure. This suggests that discomfort can be alleviated through adaptation, education or supportive measures, while intolerance may require separate management strategies to improve patient tolerance.

These results have several implications for clinical practice. Clinicians should be aware of the pain, discomfort and intolerance associated with 24-hour multichannel intraluminal impedance-pH monitoring and take appropriate measures to minimise them. Strategies may include providing adequate patient education, managing expectations and offering supportive care measures, such as topical anaesthesia when necessary. In some cases, alternative diagnostic modalities may be considered to alleviate patient discomfort during 24-hour multichannel intraluminal impedance-pH monitoring.

We previously reported that the key timing of pharyngeal reflux in patients with LPR was 2 hours post-prandially.^24^ These findings suggest that optimising the monitoring protocol, including the timing of 24-hour multichannel intraluminal impedance-pH monitoring, may be feasible. Our recent study suggests that reducing the duration of monitoring may also maintain high diagnostic sensitivity.^25^ These findings indicate that optimising both the timing and duration of 24-hour multichannel intraluminal impedance-pH monitoring could improve the detection of pharyngeal reflux events while minimising patient discomfort. In addition, improving the accuracy of simple tests, such as salivary biomarkers, would be beneficial.

Although 24-hour multichannel intraluminal impedance-pH monitoring has been reported to be uncomfortable and difficult to tolerate, most patients who underwent the test expressed satisfaction with their decision, therefore providing a sufficient explanation of the necessity of the examination and obtaining patient consent are crucial. Further research is required to reduce this discomfort.

- Fifty-five patients completed questionnaires assessing pain, discomfort and tolerance during 24-hour multichannel intraluminal impedance-pH monitoring
- All patients completed the examination, with only two minor mucosal injuries reported
- Discomfort peaked after insertion and decreased over time, whereas intolerance remained stable
- Despite discomfort, most patients reported low regret and a positive perception of the test
- Patient education and supportive care may improve acceptance and tolerance

## Supporting information

10.1017/S0022215126105192.sm001 Lee et al. supplementary material Lee et al. supplementary material

## Supplementary material

The supplementary material for this article can be found at https://doi.org/10.1017/S0022215126105192

## Table 1 Long description

Participant demographics and procedure outcomes are summarized for 55 examinations. Mean age was 55.4 years, spanning 18 to 75 years. Female participants accounted for 25 people, or 45.5 percent. Examination completion was 100 percent, with all 55 of 55 completed. Examination failure was 0 percent. Complications were limited to two mucosal injuries, indicating rare adverse events in this group.

Navigate back to Table 1.

## Table 2 Long description

Decision regret responses are reported as percentages across five statements, from strongly agree to strongly disagree. For “Regret decision,” 76 percent disagreed or strongly disagreed, while 21 percent agreed or strongly agreed. For “Did me harm,” no one strongly agreed and only 3 percent agreed; 94 percent disagreed or strongly disagreed. Positive items show more agreement than disagreement: “Right decision” has 64 percent agree or strongly agree versus 21 percent disagree or strongly disagree, and “Wise decision” has 61 percent agree or strongly agree versus 24 percent disagree or strongly disagree. “Same choice” is more mixed, with 27 percent agree or strongly agree and 70 percent disagree or strongly disagree. Percentages may not total exactly 100 for each item due to rounding.

Navigate back to Table 2.
