# Supplementary material for: Discomfort and intolerance during 24-hour multichannel intraluminal impedance-pH monitoring
Source: J Laryngol Otol. 2026 Aug;140(8):903–6. doi: 10.1017/S0022215126105192 (PMC13735490; doi:10.1017/S0022215126105192)
Supplement: Supplementary file 1 [file S0022215126105192sup001.docx]

**Supplement Table 1. Physician Questionnaire**

Patient name:

Patient age:

Patient gender:

Patient number:

Examination date:

Completion of the examination: Yes	No

Complication during the examination: Yes	No

Epistaxis	mucosal injury syncope Other complication

**Supplement Table 2. Prior to procedure questionnaire**

**<Prior to procedure>**

For the following question, please use the scale of 0-10, in which 0 is the least and 10 is the most.

How worried are you about undergoing the test?

Not at all	Very much

0	1	2	3	4	5	6	7	8	9	10

**Supplement Table 3. Immediately after the procedure questionnaire**

**<Immediately after the procedure>**

For the following question, please use the scale of 0-10, in which 0 is the least and 10 is the most.

1. How much pain did you experiencing during the examination?

Not at all	Very much

0	1	2	3	4	5	6	7	8	9	10

2. How much discomfort did you experience during the examination?

Not at all	Very much

0	1	2	3	4	5	6	7	8	9	10

3. How much were you able to tolerate during the examination?

Not at all	Very much

0	1	2	3	4	5	6	7	8	9	10

(0 points if you can't tolerate at all, and 10 points if you can tolerate without any disturbance.)

4. Is your nose in pain? Yes	No

(Please check if you answered "Yes")

Not at all	Very much

0	1	2	3	4	5	6	7	8	9	10

5. Is your throat in pain? Yes	No

(Please check if you answered "Yes")

Not at all	Very much

0	1	2	3	4	5	6	7	8	9	10

6. Do you feel any globus sensation? Yes	No

(Please check if you answered "Yes")

Not at all	Very much

0	1	2	3	4	5	6	7	8	9	10

7. Did you feel nausea? Yes	No

(Please check if you answered "Yes")

Not at all	Very much

0	1	2	3	4	5	6	7	8	9	10

8. Did you vomiting? Yes	No

(Please check if you answered "Yes")

Not at all	Very much

0	1	2	3	4	5	6	7	8	9	10

9. Did you experience difficulty breathing? Yes	No

(Please check if you answered "Yes")

Not at all	Very much

0	1	2	3	4	5	6	7	8	9	10

10. How worried are you right now?

Not at all	Very much

0	1	2	3	4	5	6	7	8	9	10

*10 points if the pain is unbearable

**Supplement Table 4. Before sleep during the monitoring**

**<Before sleep>**

For the following question, please use the scale of 0-10, in which 0 is the least and 10 is the most.

1. How much pain did you experiencing during the examination?

Not at all	Very much

0	1	2	3	4	5	6	7	8	9	10

2. How much discomfort did you experience during the examination?

Not at all	Very much

0	1	2	3	4	5	6	7	8	9	10

3. How much were you able to tolerate during the examination?

Not at all	Very much

0	1	2	3	4	5	6	7	8	9	10

**Supplement Table 5. Post procedure questionnaire**

**<Post procedure>**

For the following question, please use the scale of 0-10, in which 0 is the least and 10 is the most.

1. How much pain did you experiencing during the examination?

Not at all	Very much

0	1	2	3	4	5	6	7	8	9	10

2. How much discomfort did you experience during the examination?

Not at all	Very much

0	1	2	3	4	5	6	7	8	9	10

3. How much were you able to tolerate during the examination?

Not at all	Very much

0	1	2	3	4	5	6	7	8	9	10

4. Would you be willing to undergo another examination if necessary?

Yes	No

**Supplement Table 6. Decision Regret Scale**

1. Undergoing the 24-hr MII examination was the right decision.

a. Strongly disagree

b. Disagree

c. Neither agree nor disagree

d. Agree

e. Strongly agree

2. I regret the choice to undergo the 24-hr MII examination.

a. Strongly disagree

b. Disagree

c. Neither agree nor disagree

d. Agree

e. Strongly agree

3. I would choose to undergo the 24-hr MII examination again if I had to do it all over again.

a. Strongly disagree

b. Disagree

c. Neither agree nor disagree

d. Agree

e. Strongly agree

4. The decision to undergo 24-hr MII examination did me a lot of harm.

a. Strongly disagree

b. Disagree

c. Neither agree nor disagree

d. Agree

e. Strongly agree

5. The decision to undergo the 24-hr MII examination was a wise one.

a. Strongly disagree

b. Disagree

c. Neither agree nor disagree

d. Agree

e. Strongly agree
